# Investigation of acetyl migrations in furanosides

**DOI:** 10.1186/1860-5397-2-14

**Published:** 2006-07-21

**Authors:** O P Chevallier, M E Migaud

**Affiliations:** 1 School of Chemistry and Chemical Engineering, Queen's University, David Keir Building, Stranmillis Road, Belfast, BT9 5AG, Northern Ireland, UK

## Abstract

Standard reaction conditions for the desilylation of acetylated furanoside (riboside, arabinoside and xyloside) derivatives facilitate acyl migration. Conditions which favour intramolecular and intermolecular mechanisms have been identified with intermolecular transesterifications taking place under mild basic conditions when intramolecular orthoester formations are disfavoured. In acetyl ribosides, acyl migration could be prevented when desilylation was catalysed by cerium ammonium nitrate.

## Introduction

Over the years, a number of methods aimed at achieving chemoselective acylation of carbohydrates have been developed.[1–3] In addition to the challenges encountered in the selective acylation of compounds incorporating multiple hydroxyl groups, subsequent isomerisations of partially acylated products often arise, in particular in the case of acetyl groups.[4] Such migrations take place under varied reaction conditions, often accounting for much of yield loss[5–6] and are often proposed to involve the formation of an orthoester intermediate[7] between adjacent or remote alcohol and ester groups.[8]

We aim to prepare analogues of 1''-*O*-acetyl adenosine diphosphate ribose (AcO-ADPR) incorporating modified acetylated ribosides, xylosides and arabinosides in place of the ribose moiety. The synthesis of such compounds requires access to well-characterised acetylated xylose, arabinose and ribose synthetic precursors and while few acetylated furanosides have been reported in the literature, those that have, have often been obtained in modest yields and reliable compound characterisations have been limited.[9–11]

**Scheme 1:**
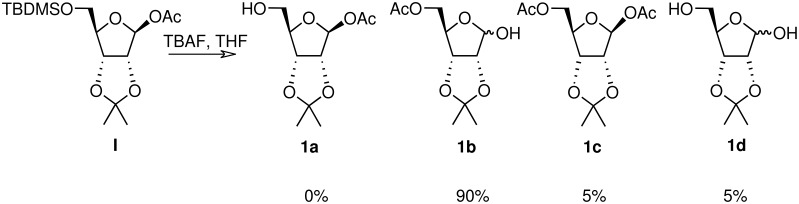
Acetyl migration products upon TBAF/THF treatment

Whilst attempting the synthesis of 1-*O*-acetyl, 2,3-isopropylidene riboside **1a** from the 5-*O*-silylated precursor **1** (Scheme 1) using mild desilylating conditions, three compounds were isolated, evidently products of acyl migration processes. In order to synthesise acetylated ADPR analogues, unequivocally, the free C5-hydroxyl derivatives of the monoacetylated furanosides must be obtained without rearrangement upon protecting group manipulation. Consequently, it was decided to carefully characterise all possible rearrangement products occurring in acetylated furanoside precursors. To the best of our knowledge, acyl migrations occurring under such mild reaction conditions in furanosides has not been investigated in contrast with the extensive work that has been carried out on pyranosides.[12–15] Therefore, an investigation on the mechanisms, reaction conditions and structural features, which control this type of chemistry in furanosides was initiated and conditions under which such migration could be minimised were sought. To this end, a series of acetylated furanosides (**1–5**) were synthesised. Product distribution due to acetyl migration was carefully examined when these compounds were treated under neutral or mildly basic/acidic reaction conditions that promote the unmasking of a silylated alcohol.

## Results and discussion

**Scheme 2:**
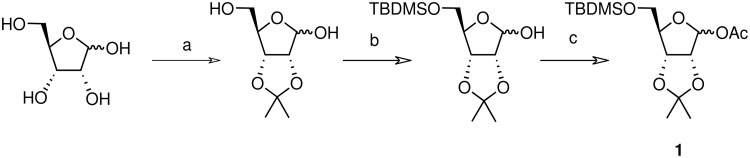
Synthesis of riboside **1**. a) 2,2-Dimethoxypropane, *p*-toluenesulfonic acid, acetone (65%); b) TBDMSCl, pyridine/DCM, DMAP (80%); c) Ac_2_O, pyridine, DMAP (90%)

All acetylated furanosides were prepared according to synthetic routes which favoured divergent syntheses (see additional information for full experimental data). Riboside **1** was synthesised from ribose in 47% overall yield via a chemoselective silylation of the C-5 primary hydroxyl group of 2,3-*O*-isopropylidene ribose (Scheme 2). The acetylated xyloside **2** and riboside **3** were both synthesised from xylose in 46% and 45% overall yield, respectively.

**Scheme 3:**
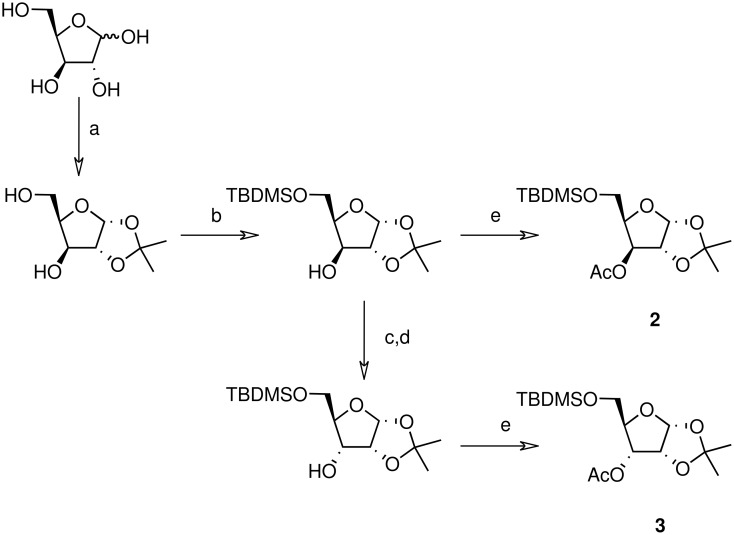
Synthesis of xyloside **2** and riboside **3**. a) i) acetone, *p*-toluenesulfonic acid, CuSO_4_; ii) HCl 0.2 M (65%); b) TBDMSCl, pyridine/DCM, DMAP (79%); c) (COCl)_2_, DMSO, Et_3_N, DCM (100%); d) NaBH_4_, THF/H_2_O (100%); e) Ac_2_O, pyridine, DMAP (90%; both **2** and **3**).

**Scheme 4:**
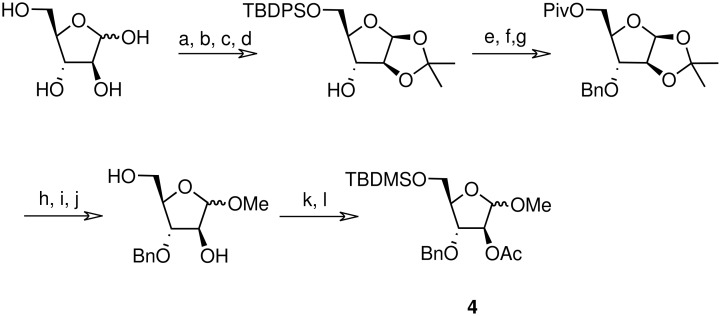
Synthesis of arabinoside **4**. a) HSEt, 6M aq HCl (85%); b) TBDPSCl, imidazole, DMAP, DMF (94%); c) HgO, HgCl_2_, acetone (80%); d) 2,2-dimethoxypropane, *p*-toluenesulfonic acid, acetone (85%); e) BnBr, NaH, THF (97%); f) TBAF, THF (87%); g) PivCl, pyridine/DCM, DMAP (85%); h) TFA/H_2_O (8/2) (87%); i) MeOH, H_2_SO_4_ (80%); j) MeONa/MeOH (76%); k) TBDMSCl, pyridine/DCM, DMAP (85%); l) Ac_2_O, pyridine, DMAP (98%).

**Scheme 5:**
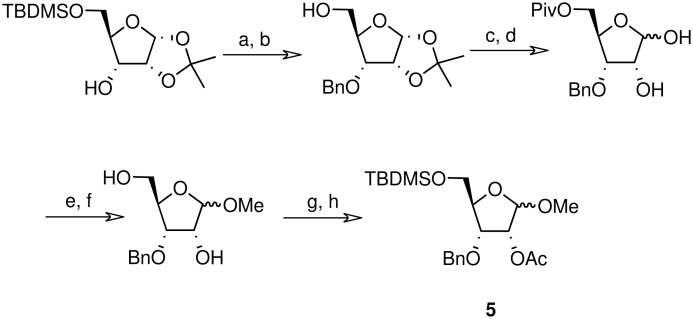
Synthesis of riboside **5**. a) BnBr, NaH, THF (82%); b) TBAF, THF (84%); c) PivCl, pyridine/DCM, DMAP (95%); d) TFA/H_2_O (8/2) (78%); e) MeOH, H_2_SO_4_ (73%); f) MeONa/MeOH (85%); g) TBDMSCl, pyridine/DCM, DMAP (78%); h) Ac_2_O, pyridine, DMAP(98%).

The methyl arabinoside **4** was synthesised from arabinose (Scheme 4) in 17% overall yield in 12 steps; a long synthetic sequence which was dictated by the preferred pyranoside form of arabinose in solution. The successful synthesis required three successive protecting group interconversions to allow for the base catalysed benzylation of the C-3 hydroxyl and the acid catalysed acetonide removal prior to acetylation at the C-2 position of the methyl arabinoside. While an acid-catalysed benzylation would have significantly shorten the route, it was found to be unsuccessful when carried out on 5-*O*-TBDPS-protected 1,2-*O*-isopropylidene arabinoside. Riboside **5** (Scheme 5) was synthesised from 5-*O*-*t*-butyldiphenylsilyl-1,2-*O*-isopropylidene arabinoside, a synthetic intermediate used in the synthesis of riboside **3**, in 12% overall yield from ribose.

During the TBAF-catalysed deprotection of the silyl ether of β-1-*O*-acetyl riboside **1**, a mixture of three different ribosyl derivatives (**1b-d**, Scheme 1) was obtained. The components of this mixture were separated by chromatography and individually identified by NMR and MS analyses. The main product **1b**, for which the anomeric proton is shielded by -0.7 ppm and the diastereotopic C-5 protons de-shielded by 0.46 and 0.53 ppm when compared with **1**, arises by an acetyl migration from the β-anomer C1-position to the C5-primary alcohol, possibly intramolecularly *via* an orthoester intermediate. Isolation of the bis-acetylated riboside **1c** and of the riboside **1d** indicated that an intermolecular trans-esterification was also taking place.

To identify the reaction conditions and the structural features of the acetylated furanosides facilitating acetyl migration upon protecting group manipulation, the various protected carbohydrates (**1–5**) were treated with tetrabutyl ammonium fluoride (TBAF) in THF, with KF in the presence of 18-crown-6 in benzene, with ceric ammonium nitrate IV (CAN) in aqueous acetonitrile and with H_2_-Pd/C in THF. No attempts were made to examine standard protic acid-catalysed desilylations such as AcOH in H_2_O/THF[16] or TFA/H_2_O in DCM[17] since we observed rapid degradation under such conditions of the synthetic intermediates of compounds **1–5**, all incorporating either an acetonide or a methyl ketal moiety.

The reactions were stopped as soon as complete disappearance of the starting material had occurred to minimise rearrangements subsequent to the deprotection step. The ratio of products and migration products were calculated based on the quantities of purified material isolated by chromatography, as ^1^H-NMRs of the crude reaction mixtures were rarely sufficient to establish such ratios accurately. The chemical shifts and the splitting patterns along with nOe experiments were sufficiently clear to allow accurate compound identification. No isomerisation was detected for any of the isolated compounds when stored in an organic solvent such as hexane, ethyl acetate or CDCl_3_ for up to 12 hrs. The chemical shifts of the ^1^H- and ^13^C-NMR of the desilylation products for compound **1** (Table 1 and Table 2) are representative of the chemical pattern for all investigated furanoside derivatives.

**Table 1:** ^1^H-NMR chemical shifts for **1** and desilylated products **1a-d**.

	H1	H2	H3	H4	H5,H5'	Others signals

**1**	6.16, s	4.68, d (J_2–3_ = 5.9 Hz)	4.77, d (J_2–3_ = 5.9 Hz)	4.31, dd (J_4–5'_ = 4.9 Hz, J_4–5_ = 8.0 Hz)	3.68, ABX (J_4–5_ = 8.1 Hz, J_5-5'_ = 10.5 Hz) 3.54, ABX (J_4–5'_ = 4.9 Hz, J_5-5'_ = 10.5 Hz)	2.03, s (CH_3_CO) 1.49, 1.33, s (CMe) 0.90, s (tBuSi) 0.09, 2xs, (MeSi)
**1a**	6.15, s	4.64, d (J_2–3_ = 6.0 Hz)	4.70, d (J_2–3_ = 6.0 Hz)	4.33, dd (J_4–5_ = J_4–5'_ = 5.4 Hz)	3.82–3.87, m	2.04, s (CH_3_CO) 1.43, 1.27, s (CMe)
**1b**	β :5.46, d (3.0 Hz) α :5.38,dd	β :4.64, d (J_2–3_ = 5.9 Hz) α : 4.65-4.63, m	β :4.70, d (J_2–3_ = 5.9 Hz) α: 4.65-4.63, m	β :4.37, dd (J_4–5_ = 7.3 Hz, J_4–5'_ = 6.0 Hz) α : 4.40-4.39, m	β :4.14, ABX (J_4–5_ = 7.3 Hz, J_5-5'_ = 11.6 Hz) β :4.07, ABX (J_4–5'_ = 6.0 Hz, J_5-5'_ = 11.6 Hz) α :4.30-4.28, m α :4.16-4.14, m	β :2.09, s (CH_3_CO) β : 1.49, 1.33, s (CMe) α :2.08, s (CH_3_CO) α : 1.57, 1.40, s (CMe)
**1c**	6.15, s	4.67, s	4.67, s	4.40, dd (J_4–5_ = 7.1 Hz; J_4–5'_ = 6.5 Hz)	4.08, ABX (J_4–5_ = 7.1 Hz, J_5-5'_ = 11.9 Hz) 4.05, ABX (J_4–5'_ = 6.5 Hz, J_5-5'_ = 11.9 Hz)	2.03, 2.00, 2xs (CH_3_CO) 1.43 1.27, s (CMe)
**1d**	β:5.34, d (6.2 Hz) α:5.44 (d, J_1–2_= 4.2 Hz, J_1-OH_= 9.8 Hz)	β:4.52, d (J_2–3_ = 5.9 Hz) α : 4.66 (dd, 1 H, J_1–2_= 4.2 Hz, J_2–3_ = 6.5 Hz)	β:4.76, d (J_2–3_ = 5.9 Hz) α : 4.74, dd (J_3–4_ = 2.0 Hz, J_2–3_ = 6.6 Hz)	β:4.31-4.27, m α: 4.31-4.27, m	β:3.67-3.64, m α:3.67-3.64, m	β:5.64 (OH_1_) β:1.43, 1.27, s (CMe) α: 1.58, 1.40, s (CMe)

**Table 2:** ^13^C-NMR chemical shifts for **1** and desilylated products **1a-d**.

	C1	C2	C3	C4	C5	Others signals

**1**	102.6	85.1	81.6	88.0	63.4	169.5 (CO) 112.7 (Cq) 26.4, 25.1 (CMe) 25.8 (SiC(CH_3_)_3_) 21.6 (CH_3_CO) 18.2 (CSi) -4.3,-4.4, ((CH_3_)_2_Si)
**1a**	102.3	85.2	80.9	88.6	63.2	169.2 (CO) 112.7 (Cq) 26.2, 24.7 (CMe) 21.0 (CH_3_CO)
**1b**	103.2 (β) 97.2 (α)	85.9 (β) 79.3 (α)	81.8 (β) 78.5 (α)	84.9(β) 81.4 (α)	65.4 (β) 64.9 (α)	170.4 (CO) 112.7 (Cβ q); 114.2 (Cα q) 26.5, 24.6 (Cβ Me); 26.0, 24.8 (Cα Me) 20.9 (CH_3_CO)
**1c**	102.0	85.2	81.4	84.9	63.9	170.2, 169.0 (CO) 113.0 (Cq) 26.2, 24.8 (CMe) 21.0, 20.6 (CH_3_CO)
**1d**	102.6 (β) 96.9 (α)	86.6 (β) 81.0 (α)	81.5 (β) 79.3 (α)	87.5 (β) 81.3 (α)	63.3 (β) 63.0 (α)	112.0 (Cβ q); 114.0 (Cα q) 26.2, 24.9 (Cβ Me); 26.2, 25.3 (Cα Me)

When derivatives **1**, **2** and **3** were reacted with a reagent source of fluoride, intramolecular acetyl migration was detected for compound **1** and **2** (**1b** and **2b**) but not for compound **3** (Table 3). The facile intramolecular migration observed for **1** and **2** involves an orthoester intermediate[18] which cannot form readily from riboside **3**. This latter observation is in contrast with the acetyl migration often reported for the glucoside series[19] and for which the orthoester intermediate (chair conformation) can form easily under mildly basic or acidic conditions. In riboside **3**, the rigid conformation of the furanose ring prevents the formation of this orthoacetate. Similarly, no migration product **4b** (Table 4) was detected when arabinoside **4** was treated with TBAF in THF while as expected, the acetyl migration between the C2 and C3 positions in the furanoside **5** occurred readily under neutral anhydrous conditions (Table 4). In riboside **5**, the five membered cyclic orthoester intermediate yields a 50-50 ratio of 2-*O* and 3-*O*-acetylated products (**5a** and **5b**, Table 4).

**Table 3:** Acetyl migration products for furanosides **1**, **2** and **3**.

	conditions	**a** R= H; R'= Ac	**b** R = Ac; R' = H	**c** R = Ac;R' = Ac	**d** R= H; R'= H

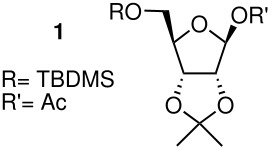		**1a**	**1b**	**1c**	**1d**
	A	0	90	5	5
	B	100	0	0	0
	C	0	70	15	15

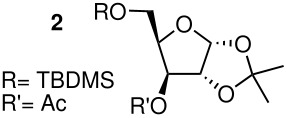		**2a**	**2b**	**2c**	**2d**
	A	5	85	5	5
	B	0	100	0	0

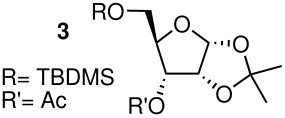		**3a**	**3b**	**3c**	**3d**
	A	90	0	5	5
	B	100	0	0	0

conditions: A: TBAF/THF; B: CAN; CH_3_CN/H_2_O; C KF. 18-crown-6; Benzene;

**Table 4:** Acetyl migration products for furanosides **4** and **5**.

	conditions	**a** R= H; R'= Ac	**b** R= Ac; R'= H	**c** R= Ac; R'= Ac	**d** R= H; R'= H

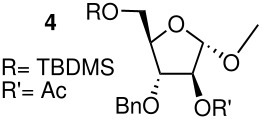		**4a**	**4b**	**4c**	**4d**
	A	90	0	5	5
	B	100	0	0	0

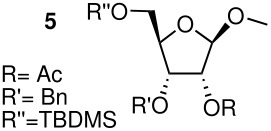		**5a**	**5b**	**5c**	**5d**
	A	90	0	5	5
	B	100	0	0	0
	
		**5e** R= H; R'= Ac R"= TBDMS	**5f** R= Ac; R'= H R"= TBDMS		
	
	D	50	50		

conditions: A: TBAF/THF; B: CAN; CH_3_CN/H_2_O; C KF. 18-crown-6; Benzene;

The formation of such an orthoester did not occur in compounds **3**, **4** or **5** under fluoride-catalysed desilylation conditions and as a result no intramolecular migration products were observed. However, for all sugars, a small proportion of bis-acetylated (**compounds c**) and non-acetylated (**compounds d**) compounds have also been isolated (Table 3 and Table 4). The proportion of intermolecular transesterification and de-esterification products increased when anhydrous KF conditions were used instead of commercial TBAF in THF (Table 3, condition C). Similarly, longer reaction time of the fluoride-catalysed desilylation reactions resulted in an increase in the formation of **c** and **d** type derivatives.

**Scheme 6:**
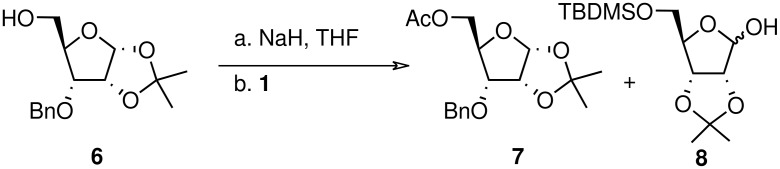
Alkoxide promoted transesterification.

In addition, the formation of the **c** and **d** furanoside derivatives took place, as easily when the liberated hydroxyl group was either *syn* or *anti* to the acetyl moiety and whether either five or six atoms separated the oxygen atoms of the silyloxy and the acetyl moieties. Such direct transesterification is intermolecular and while it has been observed in nucleoside-phosphoramidite and glycoside chemistry,[20–23] this alkoxide-promoted intermolecular acetyl migration process has been overlooked in furanosides. The isolated quantities of **1c** and **1d** decreased by a factor of two upon dilution (1:10) when **1** was reacted with TBAF, while **1b** formation remained rapid with no detection of **1a** formation. Similarly, complete transesterification took place when riboside **6** was treated with acetyl riboside **1** under basic conditions (Scheme 6).

Acetyl migration in riboside **1** was prevented when CAN was used instead of fluoride-containing reagents, indicating that the seven membered ring orthoester was not favoured under these mildly acidic conditions. This reagent was also found suitable for the silyl-removal in the other riboside **3** and **5** and arabinoside **4** with no trans-esterification occurrence. Unfortunately, the formation of a six-membered ring orthoacetate appears to be facilitated under acid-catalysed reaction conditions and total intramolecular acetyl migration occurred in xyloside **2** in the presence of CAN, showing how finally balanced things are in these systems.

In conclusion, to minimise acetyl migration in furanoside derivatives, base-catalysed reactions should be conducted under dilute conditions when the formation of an orthoacetate is disfavoured to minimise intermolecular transesterification. Unfortunately, we have been unable to identify a set of reaction conditions which could minimise the readily occurring intramolecular migration resulting from the formation of a five or six membered ring orthoacetate intermediate. Under such circumstances, migration can only be circumvented by controlling the stereochemistry of the reactive centres and opting for a multistep synthetic approach. However, it can be said that in mild acidic conditions provide better control over intra- and intermolecular acetyl migration than basic conditions. Finally, CAN has been identified as a very useful alternative reagent to TBAF in preventing unwanted base-catalysed acetyl migration in the deprotection of silylated furanosides.

## Supporting Information

File 1 contains all experimental data

## References

[1] Haines A H (1976). Adv Carbohydr Chem Biochem.

[2] Tsuda Y, Yoshimoto K (1984). Yuki Gosei Kagaku Kyokaishi.

[3] Nahmany M, Melman A (2004). Org Biomol Chem.

[4] Mastihubova M, Biely P (2004). Carbohydr Res.

[5] Orita A, Hamada Y, Nakano T, Toyoshima S, Otera J (2001). Chem–Eur J.

[6] Ishido Y, Sakairi N, Sekiya M, Nakazaki N (1981). Carbohydr Res.

[7] Dohrshuck A (1952). J Am Chem Soc.

[8] Bonner W A (1959). J Org Chem.

[9] Ning J, Kong F (2001). Carbohydr Res.

[10] Hennen W J, Sweers H M, Wang Y-F, Wong C-H (1988). J Org Chem.

[11] Moravcova J, Kefurt K, Hladuvkova R, Stanek J (2000). Collect Czech Chem Commun.

[12] Petrovic V, Tomic C, Matanovic M (2002). Carbohydr Res.

[13] Hanefeld U (2003). Org Biomol Chem.

[14] La Ferla B (2002). Monatsh Chem.

[15] Graziani A, Passacantilli P, Piancatelli G, Tani S (2001). Tetrahedron Lett.

[16] Kawai A, Hara O, Hamada Y, Shiari T (1988). Tetrahedron Lett.

[17] Baker R, Cummings W G, Hayes J F, Kumar A (1986). J Chem Soc, Chem Commun.

[18] Bouchra M, Calinaud P, Gelas J (1995). Synthesis.

[19] Horrobin T, Tran C H, Crout D (1998). J Chem Soc, Perkin Trans 1.

[20] Isai S V, Usol'tsev A A, Stiba E N (2003). Chem Nat Compd.

[21] Dickinson R G, King A R (1991). Biochem Pharmacol.

[22] Poijarvi P, Oivanen M, Lonnberg H (2001). Nucleosides, Nucleotides Nucleic Acids.

[23] Vesely J, Ledvina M, Jindrich J (2004). Collect Czech Chem Commun.

[24] Wilcox C S, Otoski R M (1986). Tetrahedron Lett.

[25] Sasaki S, Yamauchi H, Nagatsugi F, Takahashi R, Taniguchi Y, Maeda M (2001). Tetrahedron Lett.

[26] Logue M W, Han B-H (1983). Carbohydr Res.

[27] Kloosterman M, Mosmuller E W J, Shoemaker H E (1987). Tetrahedron Lett.

[28] Ishido Y, Sakairi N, Sekiya M, Nakazaki N (1981). Carbohydr Res.

[29] Chittenden G (1972). Carbohydr Res.

