# Supplementary material for: Investigation of acetyl migrations in furanosides
Source: Beilstein J Org Chem. 2006 Jul 21;2:14. doi: 10.1186/1860-5397-2-14 (PMC1592296; doi:10.1186/1860-5397-2-14)
Supplement: File 1 — contains all experimental data [file Beilstein_J_Org_Chem-02-14-s001.doc]

# Investigation of acetyl migrations in furanosides

O.P. Chevallier, M. E. Migaud*

School of Chemistry and Chemical Engineering, Queen’s University

David Keir Building, Stranmillis Road, Belfast, BT9 5AG; Northern Ireland

**Experimental**

**General procedure**

Chemicals were purchased from Sigma-Aldrich Chemical Company, Lancaster or ACROS. Solvents for extractions and chromatography were technical grade. Solvents used in reactions were freshly distilled from appropriated drying agents before use. All other reagents were recrystallised or distilled as necessary. All reactions requiring anhydrous or inert conditions were carried out in oven dried glassware under a positive atmosphere of argon. Solutions or liquids were introduced using oven dried syringes or cannula through rubber septa. All reactions were stirred magnetically using Teflon-coated stirs bars. Removal of solvents was accomplished using a rotary evaporator at water aspirator pressure or under high vacuum (0.5 mm Hg). Analytical TLC was performed with Merck Silica gel 60 F_254_ plates. Visualisation was accomplished by UV-light (λ = 254 nm) and / or staining with an anisaldehyde solution, followed by heating. ^1^H, ^13^C and 2D (H-COSY, HMQC) NMR spectra were all recorded on Brüker avance DPX 300 and Brüker avance DRX 500. Mass spectra were recorded on a VG Autospec spectrometer. Two different batches of TBAF (Aldrich n^o^ 13611TB, Alfa Aesar n^o^ 10109183) were used to carry out identical experiments in order to check the reproducibility of the results as a function of the source of the reagent.

**General procedure for the TBDMS-protection of the furanosyl C-5 hydroxyls.**

Typical procedure:

Preparation of 5-*O*-*tert*-butyldimethylsilyl-1,2-*O*-isopropylidene-α-D-xylofuranoside:

To a solution of 1,2-*O*-isopropylidene-α-D-xylofuranoside (5.02 g, 26.8 mmol) dissolved in dry pyridine/dry dichloromethane (40ml/40ml) was added dropwise a solution of *tert*-butyldimethylsilyl chloride (4.45 g, 29.5 mmol) and 4-dimethylaminopyridine (327 mg, 2.6 mmol) in dry dichloromethane (50 ml) at 0^o^C. The resulting solution was warmed up to room temperature and stirred for 4 hours. The solvent was then removed under vacuum. The white residue was dissolved in chloroform (50 ml), washed with a solution of saturated sodium hydrogen carbonate (100 ml), extracted with chloroform (3x50 ml), washed with water, brine, dried over MgSO_4_, filtered and concentrated under vacuum to afford the crude 5-*O*-*tert*-butyldimethylsilyl-1,2-*O*-isopropylidene-α-D-xylofuranoside as an oil.

Purification by flash chromatography (75/25 v/v hexane/ethyl acetate) gave 5-*O*-*tert*-butyldimethylsilyl-1,2-*O*-isopropylidene-α-D-xylofuranoside (6.51 g, 79%) as a colourless oil.

**General procedure for the protection of hydroxyl group with a pivaloate ester:**

Typical procedure:

Preparation of 1,2-*O*-isopropylidene-5-*O*-pivaloyl-α-D-xylofuranoside

1,2-*O*-isopropylidene-α-D-xylofuranoside (12.0 g, 63.1 mmol) was dissolved in dry pyridine/dry dichloromethane (50ml/50ml). To this solution was added dropwise pivaloic chloride (9.3 ml, 75.7 mmol) dissolved in dry dichloromethane (40 ml) at 0^o^C. The resulting solution was kept under stirring and allowed to warm up at room temperature for 4 hours. The solvent was then removed under vacuum. The white residue was dissolved in chloroform (100 ml), washed with a solution of saturated sodium hydrogen carbonate (100 ml), washed with water, brine, dried, filtered and concentrated under vacuum to afford the crude as an oil.

Purification by flash chromatography (gradient 85/15 to 65/35 v/v hexane/ethyl acetate) gave 1,2-*O*-isopropylidene-5-*O*-pivaloyl-α-D-xylofuranoside (15.2 g, 88%) as a colourless oil.

**General procedure for benzylation:**

Typical procedure:

Preparation of 3-*O*-benzyl-5-*O*-*tert*-butyldiphenylsilyl-1,2-*O*-isopropylidene-β-D-arabino-furanoside

To a solution of 5-*O*-*tert*-butyldiphenylsilyl-1,2-*O*-isopropylidene-β-D-arabinofuranoside (1.62 g, 3.78 mmol) in dry THF (30 ml) was added benzyl bromide (0.53 ml, 4.54 mmol) and sodium hydride (181 mg, 4.60 mmol) at 0^o^C. The resulting reaction mixture was allowed to warm up at room temperature and was kept under stirring overnight. After addition of diethyl ether (50 ml), the organic fraction was washed with a solution of saturated NH_4_Cl (50 ml), water, brine, dried, filtered and concentrated under vacuum to afford the crude as a yellow oil.

Purification by flash chromatography (9/1 v/v petroleum ether/ethyl acetate) gave 3-*O*-benzyl-5-*O*-*tert*-butyldiphenylsilyl-1,2-*O*-isopropylidene-β-D-arabinofuranoside (1.91g, 97%) as a yellow oil.

**General procedure for acetylation:**

Typical procedure:

Preparation of 3-*O*-acetyl-5-*O*-*tert*-butyldimethylsilyl-1,2-*O*-isopropylidene-α−D-ribofura-noside.

To a solution of 5-*O*-*tert*-butyldimethylsilyl-1,2-*O*-isopropylidene-α−D-ribofuranoside (3.98 g, 13.1 mmol) and 4-dimethylaminopyridine (158 mg, 1.3 mmol) in pyridine (35 ml) was added acetic anhydride (2.5 ml, 26.3 mmol). The resulting solution was stirred overnight at room temperature. Then the reaction mixture was concentrated under vacuum to afford 3-*O-*acetyl-5-*O*-*tert*-butyldimethylsilyl-1,2-*O*-isopropylidene-α−D-ribofuranoside (4.5 g, 100%) as a yellow oil.

**General procedure for the Fisher reaction:**

Typical procedure:

Preparation of methyl 3-*O*-benzyl-5-*O*-pivaloyl-D-ribofuranoside

To a solution of 3-*O*-benzyl-5-*O*-pivaloyl−D-ribofuranose (380 mg, 1.17 mmol) in MeOH (20 ml) was added H_2_SO_4_ (0.5 ml). The reaction mixture was then kept under stirring for 2 hours before been diluted with EtOAc (50 ml) and neutralized with a saturated solution of Na_2_CO_3_. The resulting mixture was extracted with EtOAc (3x20 ml), combined organics layers were washed with water, brine, dried, filtered and concentrated under vacuum to afford an oil.

Purification by flash chromatography (1/1 v/v petroleum ether/ethyl acetate) yielded methyl 3-*O*-benzyl-5-*O*-pivaloyl-D-ribofuranoside as a mixture α/β (25/75) (361 mg, 73%) as a colourless oil.

**General procedure for the deprotection of a silyl ether derivative using TBAF:**

Typical procedure:

Preparation of 3-*O*-benzyl-1,2-*O*-isopropylidene-α−D-ribofuranoside

3-*O*-benzyl-5-*O*-*tert*-butyldimethylsilyl-1,2-*O*-isopropylidene-α−D-ribofuranoside (1.21 g, 3.07 mmol) was dissolved in THF (30 ml). To this resulting solution was added TBAF (3.6 ml. 3.60 mmol) and was left under stirring at room temperature for 4 hours. A saturated solution of NH_4_Cl (50 ml) was then added, aqueous layer was extracted with chloroform (3x25 ml), combined organics layers were washed with brine, dried, filtered and concentrated under vacuum to afford an oil.

Purification by flash chromatography (6/4 v/v petroleum ether/ethyl acetate) afforded 3-*O*-benzyl-1,2-*O*-isopropylidene-α−D-ribofuranoside (720 mg, 84%) as a colourless oil.

**General procedure for the deprotection of a silyl ether derivative using CAN:**

Typical procedure:

Preparation of methyl 2-*O*-acetyl-3-*O*-benzyl-α-D-arabinofuranoside

To a solution of methyl 2-*O*-acetyl-3-*O*-benzyl-5-*O*-*tert*-butyldimethylsilyl-α-D-arabinofuranoside (21.6 mg, 0.05 mmol) dissolved in a mixture of acetonitrile/water (9ml/1ml) was added CAN (35 mg, 0.06 mmol). The resulting reaction mixture was stirred at room temperature for 4 hours. A saturated solution of NaHCO_3_ was then added, aqueous layer was extracted with chloroform (20 ml; 3x), combined organics layers were washed with brine, dried, filtered and concentrated under vacuum to afford methyl 2-*O*-acetyl-3-*O*-benzyl-α-D-arabinofuranoside (14.8 mg, 95%) as a yellow oil.

**General procedure for the deprotection of a pivaloate ester:**

Typical procedure:

Preparation of methyl 3-*O*-benzyl-β-D-ribofuranoside

Methyl 3-*O*-benzyl-5-*O*-pivaloyl−D-ribofuranoside (200 mg, 0.58 mmol) was dissolved in MeOH (10 ml). To the resulting solution was added MeONa (0.01 g) and was left under stirring for 2 hours. The solution was then diluted with EtOAc (10 ml) and a solution of NH_4_Cl was added. Aqueous layer was extracted with EtOAc (3x10 ml) combined organics layers were washed with water, brine, dried, filtered and concentrated under vacuum to afford the crude as a colourless oil.

Purification by flash chromatography (gradient from 1/1 to 3/7 v/v petroleum ether/ethyl acetate) yielded methyl 3-*O*-benzyl-β-D-ribofuranoside (109 mg, 73%) and methyl 3-*O*-benzyl-α-D-ribofuranoside (34 mg, 14%).

**General procedure for the deprotection of an isopropylidene:**

Typical procedure:

Preparation of 3-*O*-benzyl-5-*O*-pivaloyl-D-arabinofuranose:

3-*O*-benzyl-1,2-*O*-isopropylidene-5-*O*-pivaloyl-β-D-arabinofuranoside (898 mg, 2.46 mmol) was treated with a mixture TFA/H_2_O (18 ml/2ml) at 0^O^C. The resulting solution was left under stirring for 3 hours and was then diluted with EtOAc. A saturated solution of Na_2_CO_3_ was added and Na_2_CO_3_ solid was added until the pH was neutral. Aqueous layer was extracted with EtOAc (3x30 ml), combined organic layers were washed with water, brine, dried, filtered and concentrated under vacuum to afford the deprotected carbohydrate as oil.

Purification by flash chromatography (1/1 v/v petroleum ether/ethyl acetate) gave 3-*O*-benzyl-5-*O*-pivaloyl-D-arabinofuranose (695 mg, 87%) as a α/β mixture (2/3).

**General procedure for debenzylation:**

Methyl 2-*O*-acetyl-3-*O*-benzyl-5-*O*-*tert*-butyldimethylsilyl-β-D-ribofuranoside (160 mg, 0.39mmol) was diluted in THF (20 ml) at room temperature and added under argon to non-pre-treated commercial Pd/C (10%). The pH of the reaction solution was measured to be at 6 for one given batch of catalyst. The mixture was stirred under H_2_ pressure (balloon pressure) overnight. The catalyst was then filtered off (AUTOTOP syringe filters, Whatman) and the solvent removed under reduced pressure. The crude was then purified by flash chromatography (1/1 v/v petroleum ether/ethyl acetate) to yield quantitatively methyl 2-*O*-acetyl-5-*O*-*tert*-butyldimethylsilyl-β-D-ribofuranoside and methyl 3-*O*-acetyl-5-*O*-*tert*-butyldimethylsilyl-β-D-ribofuranoside as a mixture of regioisomers.

**3.12. Characterisation**

**1**-***O-*acetyl-5-*O*-*tert*-butyldimethylsilyl-2,3-*O*-isopropylidene-β-D-ribofuranoside 1**^24^

overall yield from ribose: 55.6 % after 3 steps

[α]_D_^20^= -55.4^o^ (c=1.3, CHCl_3_); HRMS *m/z* 364.214828 (calcd for C_16_H_34_O_6_NSi([M+NH_4_]^+^) 364.215542).

**1-*O*-acetyl-2,3-*O*-isopropylidene-β-D-ribofuranoside 1a**

[α]_D_^20^= -57.4^o^ (c=2.1, CHCl_3_); HRMS *m/z* 250.129498 (calcd for C_10_H_20_O_6_N([M+NH_4_]^+^) 250.129063).

**5-*O*-acetyl-2,3-*O*-isopropylidene-D-ribofuranoside 1b**^25^

HRMS *m/z* 250.129032 (calcd for C_10_H_20_O_6_N([M+NH_4_]^+^) 250.129063).

**1,5-*O*-acetyl-2,3-*O*-isopropylidene-β-D-ribofuranoside 1c**^26^

[α]_D_^20^= -56.7^o^ (c=2.0, CHCl_3_); HRMS *m/z* 292.138962 (calcd for C_12_H_22_O_7_N ([M+NH_4_]^+^) 292.139627).

**3-*O*-acetyl-5-*O*-*tert*-butyldimethylsilyl-1,2-*O*-isopropylidene-α-D-xylofuranoside 2**

Overall yield from xylose: 46.2% after 3 steps

^1^H NMR (CDCl_3_) δ 5.90 (d, J_1,2_= 3.8 Hz, 1H, H_1_); 5.24 (d, J_3,4_= 3.0 Hz, 1H, H_3_); 4.50 (d, J_1,2_= 3.7 Hz, 1H, H_2_); 4.36 (ddd, J_3,4_=3.1 Hz, J_4,5’_= 5.9 Hz, J_4,5_=7.6 Hz, 1H, H_4_); 3.85(ABX, J_4,5_=7.6 Hz, J_5,5’_=10.0 Hz, 1H, H_5_); 3.79 (ABX, J_4,5_=5.5 Hz, J_5,5’_=10.0 Hz, 1H, H_5’_); 2.07 (s, 3H, CH_3Ac_); 1.49 (s, 3H, CH_3_); 1.21 (s, 3H, CH_3_); 0.83 (s, 9H, tBu); 0.03 (s, 6H, CH_3_). ^13^C NMR (CDCl_3_) δ 169.6 (C_OAc_); 112.7 (C_q_); 104.8 (C_1_); 83.2 (C_2_); 79.2 (C_4_); 75.9 (C_3_); 59.9 (C_5_); 26.7(C_Me_); 26.3 (C_Me_); 25.7 (C_tBu_); 20.7 (C_MeAc_); 18.2 (C_Si_); -5.4 (C_MeSi_); -5.6 (C_MeSi_). [α]_D_^20^= -12.1^o^ (c=0.1, CHCl_3_); HRMS *m/z* 347.188820 (calcd for C_16_H_31_O_6_Si([M+] 347.188992).

**3-*O*-acetyl-1,2-*O*-isopropylidene-α-D-xylofuranoside 2a**^27^

^1^H NMR (CDCl_3_) δ 5.92 (d, J_1,2_=3.6 Hz, 1H, H_1_); 5.19 (d, J_3,4_=2.7 Hz, 1H, H_3_); 4.56 (d, J_1,2_=3.6 Hz, 1H, H_2_); 4.38 (ddd, J_3,4_=2.8 Hz, J_4,5_=6.0 Hz, J_4,5’_= 6.0 Hz, 1H, H_4_); 3.82 (ABX, J_4,5_=6.0 Hz, J_5,5’_=12.0 Hz, 1H, H_5_); 3.62 (ABX, J_4,5’_=6.0 Hz, J_5,5’_=12.0 Hz, 1H, H_5’_); 2.11 (s, 3H, CH_3Ac_); 1.52 (s, 3H, CH_3_); 1.32 (s, 3H, CH_3_). ^13^C NMR (CDCl_3_) δ 170.6 (C_OAc_); 112.2 (C_qIso_); 104.5 (C_1_); 83.5 (C_2_); 79.4 (C_4_); 76.5 (C_3_); 59.6 (C_5_); 26.6 (C_MeIso_); 26.2 (C_MeIso_); 20.7 (C_MeAc_).

**5-*O*-acetyl-1,2-*O*-isopropylidene-α−D-xylofuranoside 2b**^28^

^1^H NMR (CDCl_3_) δ 5.92 (d, J_1,2_= 3.6 Hz, 1H, H_1_); 4.56 (d, J_1,2_= 3.6 Hz, 1H, H_2_); 4.53 (ABX, J_4,5_=7.5 Hz, J_5,5’_=11.6 Hz, 1H, H_5_); 4.26 (ddd, J_3,4_=2.5 Hz, J_4,5’_=5.1 Hz, J_4,5_= 7.5 Hz, 1H, H_4_); 4.16 (ABX, J_4,5’_=5.1 Hz, J_5,5’_=11.6 Hz, 1H, H_5’_); 4.12 (dd, J_3,4_= 2.8 Hz, J_2,3_= 4.3 Hz, 1H, H_3_); 2.86 (br, 1H, OH); 2.11 (s, 3H, CH_3Ac_); 1.51 (s, 3H, CH_3_); 1.32 (s, 3H, CH_3_).

^13^C NMR (CDCl_3_) δ 171.9 (C_OAc_); 111.9 (C_q_); 104.7 (C_1_); 85.0 (C_3_); 78.2 (C_2_); 74.5 (C_4_); 61.0 (C_5_); 26.8(C_Me_); 26.2 (C_Me_); 20.8 (C_MeAc_). HRMS *m/z* 250.128128 (calcd for C_10_H_20_O_6_N([M+NH_4_]^+^) 250.129063).

**3,5-*O*-acetyl-1,2-*O*-isopropylidene-α-D-xylofuranoside 2c**^5^

^1^H NMR (CDCl_3_) δ 5.92 (d, J_1,2_= 3.7 Hz, 1H, H_1_); 5.23 (d, J_3,4_= 3.0 Hz, 1 H, H_3_); 4.50 (d, J_1,2_= 3.8 Hz, 1H, H_2_); 4.47 (ddd, J_3,4_=3.1 Hz, J_4,5’_=4.9 Hz, J_4,5_=7.7 Hz, 1H, H_4_); 4.26 (ABX, J_4,5_=7.3 Hz, J_5,5’_=11.6 Hz, 1H, H_5_); 4.16 (ABX, J_4,5’_=4.9 Hz, J_5,5’_=11.6 Hz, 1H, H_5’_); 2.07, 2.05 (s, 6H, CH_3Ac_); 1.50 (s, 3H, CH_3_); 1.29 (s, 3H, CH_3_). ^13^C NMR (CDCl_3_) δ 170.5, 169.6 (C_OAc_); 112.2 (C_qIso_); 104.8 (C_1_); 83.2 (C_2_); 76.6 (C_4_); 76.0 (C_3_); 61.3 (C_5_); 26.6 (C_MeIso_); 26.1 (C_MeIso_); 20.7, 20.6 (C_MeAc_). [α]_D_^20^= -3.6^o^ (c=3.0, CHCl_3_) HRMS *m/z* 292.139038 (calcd for C_12_H_22_O_7_N([M+NH_4_]^+^) 292.139627).

**3-*O*-acetyl-5-*O*-*tert*-butyldimethylsilyl-1,2-*O*-isopropylidene-α-D-ribofuranoside 3**

Overall yield from xylose: 41.2% after 5 steps

^1^H NMR (CDCl_3_) δ 5.77 (d, J_1,2_= 2.9 Hz, 1H, H_1_); 4.77 (m, 2H, H_3_, H_2_); 4.13 (ddd, J_4,5’_=2.7 Hz, J_4,5_=2.7 Hz, J_3,4_=6.1 Hz, 1H, H_4_); 3.83 (ABX, J_4,5_=2.8 Hz, J_5,5’_=11.9 Hz, 1H, H_5_); 3.69 (ABX, J_4,5’_=2.3 Hz, J_5,5’_=11.9 Hz, 1H, H_5’_); 2.04 (s, 3H, CH_3Ac_); 1.46 (s, 3H, CH_3_); 1.25 (s, 3H, CH_3_); 0.86 (s, 9H, tBu); 0.04, 0.04 (s, 6H, CH_3_). ^13^C NMR (CDCl_3_) δ 169.9 (C_OAc_); 112.6 (C_qIso_); 104.1 (C_1_); 78.6 (C_2_); 77.5 (C_4_); 71.9 (C_3_); 61.3 (C_5_); 26.5 (C_MeIso_); 26.4 (C_MeIso_); 20.4 (C_MeAc_); 18.0 (C_Si_); -5.0, -5.4 (C_MeSi_). HRMS *m/z* 347.188132 (calcd for C_16_H_31_O_6_Si([M+] 347.188992).

**3-*O*-acetyl-1,2-*O*-isopropylidene-α-D-ribofuranoside 3a**

^1^H NMR (CDCl_3_) δ 5.75 (d, J_1,2_= 3.6 Hz, 1H, H_1_); 4.78 (m, 2H, H_2_, H_3_); 4.12 (m, 1H, H_4_); 3.82 (ABX, J_4,5_=3.1 Hz, J_5,5’_=12.9 Hz, 1H, H_5_); 3.55 (ABX, J_4,5’_=2.2 Hz, J_5,5’_=12.9 Hz, 1H, H_5’_); 2.06 (s, 3H, CH_3Ac_); 1.50 (s, 3H, CH_3_); 1.29 (s, 3H, CH_3_). ^13^C NMR (CDCl_3_) δ 170.1 (C_OAc_); 112.7 (C_qIso_); 103.7 (C_1_); 77.9 (C_2_); 76.7 (C_4_); 71.2 (C_3_); 60.2 (C_5_); 26.5 (C_MeIso_); 26.2 (C_MeIso_); 20.2 (C_MeAc_).

**5-*O*-acetyl-1,2-*O*-isopropylidene-α-D-ribofuranoside 3b**

^1^H NMR (CDCl_3_) δ 5.83 (d, J_1,2_= 3.8 Hz, 1H, H_1_); 4.59 (dd, J_1,2_= 3.8 Hz, J_2,3_= 4.9 Hz, 1H, H_2_); 4.44 (ABX, J_4,5’_= 5.7 Hz, J_5,5’_= 12.3 Hz, 1H, H_5’_); 4.19 (ABX, J_4,5_= 2.4 Hz, J_5,5’_= 12.3 Hz, 1H, H_5_); 3.95 (ddd, J_4,5_= 2.4 Hz, J_4,5’_= 5.7 Hz, J_3.4_= 8.4 Hz, 1H, H_4_); 3.87-3.82 (m, 1H, H_3_); 2.41 (br, J= 10.5 Hz, 1H, OH); 2.10 (s, 3H, CH_3_OAc); 1.58 (s, 3H, CH_3_); 1.38 (s, 3H, CH_3_). ^13^C NMR (CDCl_3_) δ 170.9 (C_OAc_); 112.8 (C_qIso_); 104.0 (C_1_); 78.3 (C_4_); 78.2 (C_2_); 72.0 (C_3_); 63.0 (C_5_); 26.5 (C_MeIso_); 26.4 (C_MeIso_); 20.8 (C_MeAc_).

**3,5-*O*-acetyl-1,2-*O*-isopropylidene-α-D-ribofuranoside 3c**^29^

^1^H NMR (CDCl_3_) δ 5.81 (d, J_1,2_= 3.8 Hz, 1H, H_1_); 4.80 (dd, J_1.2_= 3.8 Hz, J_2,3_= 4.8 Hz, 1H, H_2_); 4.66 (dd, J_2,3_= 4.8 Hz, J_3,4_= 9.2 Hz, 1 H, , H_3_); 4.29 (ddd, J_4,5’_=2.5 Hz, J_4,5_=5.3 Hz, J_3,4_=9.1 Hz, 1H, H_4_); 4.34 (ABX, J_4,5_=5.0 Hz, J_5,5’_=12.3 Hz, 1H, H_5_); 4.12 (ABX, J_4,5’_=2.5 Hz, J_5,5’_=12.3 Hz, 1H, H_5’_); 2.11, 2.07 (s, 6H, CH_3Ac_); 1.54 (s, 3H, CH_3_); 1.32 (s, 3H, CH_3_). ^13^C NMR (CDCl_3_) δ 170.6, 170.1 (C_OAc_); 113.1 (C_qIso_); 104.1 (C_1_); 77.1 (C_2_); 75.3 (C_4_); 72.1 (C_3_); 62.3 (C_5_); 26.5 (C_MeIso_); 26.5 (C_MeIso_); 20.7, 20.6 (C_MeAc_). [α]_D_^20^=80.6^o^ (c=1.1, CHCl_3_) HRMS *m/z* 292.139969 (calcd for C_12_H_22_O_7_N([M+NH_4_]^+^) 292.139627).

**Methyl 2-*O*-acetyl-3-*O*-benzyl-5-*O*-*tert*-butyldimethylsilyl-α-D-arabinofuranoside 4**

Overall yield from arabinose: 8.3% after 12 steps

^1^H NMR (CDCl_3_) δ 7.33-7.28 (m, 5H, H_Ar_); 5.10 (d, J_2.3_= 1.2 Hz, 1H, H_2_); 4.89 (s, 1H, H_1_); 4.72 (AB, J_A_= 12.2 Hz, 1H, H_CH2_); 4.59 (AB, J_A_= 12.2 Hz, 1H, H_CH2_); 4.11 (dd, J_4,5_= 4.4 Hz, J_3.4_= 9.4 Hz, 1H, H_4_); 3.89-3.87 (m, 1H, H_3_); 3.73 (ABX, J_4,5_= 4.5 Hz, J_5,5’_= 11.3 Hz, 1H, H_5_); 3.71 (ABX, J_4,5’_= 4.2 Hz, J_5,5’_= 11.3 Hz, 1H, H_5’_); 3.39 (s, 3H, H_OMe_); 2.06 (s, 3H, CH_3Ac_); 0.87 (s, 9H, tBu); 0.04, 0.02 (s, 6H, CH_3_).

^13^C NMR (CDCl_3_) δ 169.9(C_OAc_); 137.7 (C_Ar_); 128.3, 127.9, 127.7 (C_Ar_); 107.0 (C_1_); 83.6 (C_4_); 82.8 (C_3_); 81.7 (C_2_); 72.1 (C_CH2_); 62.5 (C_5_); 54.7 (C_Me_); 25.8 (C_tBu_); 20.9 (C_MeAc_); 18.3 (C_Si_); -5.3, -5.4 (C_MeSi_).

**Methyl 2-*O*-acetyl-3-*O*-benzyl-α-D-arabinofuranoside 4a**

^1^H NMR (CDCl_3_) δ 7.34-7.28 (m, 5H, H_Ar_); 5.10 (d, J= 1.0 Hz, 1H, H_2_); 4.90 (s, 1H, H_1_); 4.76 (AB, J_A_= 12.2 Hz, 1H, H_CH2_); 4.58 (AB, J_A_= 12.2 Hz, 1H, H_CH2_); 4.15 (m, 1H, H_4_); 3.89-3.87 (m, 1H, H_3_); 3.83 (ABX, J_4,5_= 3.0 Hz, J_5,5’_= 11.9 Hz, 1H, H_5_); 3.58 (ABX, J_4,5’_= 2.8 Hz, J_5,5’_= 11.9 Hz, 1H, H_5’_); 3.40 (s, 3H, H_OMe_); 2.07 (s, 3H, CH_3Ac_). ^13^C NMR (CDCl_3_) δ 169.8(C_OAc_); 137.6 (C_Ar_); 128.4, 127.9, 127.8 (C_Ar_); 107.2 (C_1_); 83.1 (C_4_); 82.6 (C_3_); 81.6 (C_2_); 72.4 (C_CH2_); 61.9 (C_5_); 54.9 (C_Me_); 20.9 (C_MeAc_).

HRMS *m/z* 314.161613 (calcd for C_15_H_24_O_6_N ([M+NH_4_]^+^) 314.160363).

**Methyl 2-*O*-acetyl-3-*O*-benzyl-5-*O*-*tert*-butyldimethylsilyl-β-D-ribofuranoside 5**

Overall yield from xylose: 10.3% after 12 steps

^1^H NMR (CDCl_3_) δ 7.33-7.28 (m, 5H, H_Ar_); 5.18 (d, J_2,3_= 4.5 Hz, 1H, H_2_); 4.87 (s, 1H, H_1_); 4.57 (AB, J_A_= 11.4 Hz, 1H, H_CH2_); 4.45 (AB, J_A_= 11.4 Hz, 1H, H_CH2_); 4.18 (dd, J_2.3_= 4.6 Hz, J_3.4_= 7.2 Hz, 1H, H_3_); 4.08 (m, 1H, H_4_); 3.74 (ABX, J_4,5_= 4.7 Hz, J_B_= 11.0 Hz, 1H, H_5_); 3.65 (ABX, J_4,5’_= 3.8 Hz, J_5,5’_= 11.0 Hz, 1H, H_5’_); 3.34 (s, 3H, H_OMe_); 2.11 (s, 3H, CH_3Ac_); 0.90 (s, 9H, tBu); 0.06 (s, 6H, CH_3_). ^13^C NMR (CDCl_3_) δ 170.1 (C_OAc_); 137.7 (C_Ar_); 128.4, 127.9 (C_Ar_); 106.0 (C_1_); 82.1 (C_4_); 77.2 (C_3_); 74.4 (C_2_); 73.0 (C_CH2_); 63.6 (C_5_); 55.0 (C_Me_); 25.9 (C_tBu_); 20.9 (C_MeAc_); 18.3 (C_Si_); -5.3, -5.4 (C_MeSi_). [α]_D_^20^= -1.8^o^ (c=1.5, CHCl_3_) HRMS *m/z* 428.246651 (calcd for C_21_H_38_O_6_NSi ([M+NH_4_]^+^) 428.246842).

**Methyl 2-*O*-acetyl-3-*O*-benzyl-β-D-ribofuranoside 5a**

^1^H NMR (CDCl_3_) δ 7.34-7.29 (m, 5H, H_Ar_); 5.19 (d, J_2,3_= 4.5 Hz, 1H, H_2_); 4.87 (s, 1H, H_1_); 4.58 (AB, J_A_= 11.4 Hz, 1H, H_CH2_); 4.45 (AB, J_A_= 11.4 Hz, 1H, H_CH2_); 4.24 (dd, J_2.3_= 4.5 Hz, J_3.4_= 7.5 Hz, 1H, H_3_); 4.17-4.14 (m, 1H, H_4_); 3.79 (ABX, J_4,5_= 4.4 Hz, J_5,5’_= 11.3 Hz, 1H, H_5_); 3.56 (ABX, J_4,5’_= 1.7 Hz, J_5,5’_= 11.3 Hz, 1H, H_5’_); 3.40 (s, 3H, H_OMe_); 2.12 (s, 3H, CH_3Ac_). ^13^C NMR (CDCl_3_) δ 170.0 (C_OAc_); 137.4 (C_Ar_); 128.4, 128.0, 127.9 (C_Ar_); 106.6 (C_1_); 82.1 (C_4_); 76.4 (C_3_); 74.2 (C_2_); 73.2 (C_CH2_); 62.3 (C_5_); 55.6 (C_Me_); 20.8 (C_MeAc_). HRMS *m/z* 314.160812 (calcd for C_15_H_24_O_6_N([M+NH_4_]^+^) 314.160363).

**Methyl 2,5-*O*-acetyl-3-*O*-benzyl-β-D-ribofuranoside 5c**

^1^H NMR (CDCl_3_) δ 7.34-7.29 (m, 5H, H_Ar_); 5.21 (d, J_2,3_= 4.3 Hz, 1H, H_2β_); 4.86 (s, 1H, H_1β_); 4.60 (AB, J_A_= 11.4 Hz, 1H, H_CH2β_); 4.43 (AB, J_A_= 11.4 Hz, 1H, H_CH2β_); 4.21 (ddd, J_4,5’_= 3.1 Hz, J_4,5_= 5.7 Hz, J_3.4_= 7.9 Hz, 1H, H_4β_); 4.29 (ABX, J_4,5_= 5.7 Hz, J_5,5’_= 11.7 Hz, 1H, H_5_); 4.06 (ABX, J_4,5’_= 3.2 Hz, J_5,5’_= 11.7 Hz, 1H, H_5’_);4.12 (dd, 1H, J_2.3_= 4.3 Hz, J_3.4_= 7.9 Hz, 1H, H_3β_); 3.34 (s, 3H, H_OMe_); 2.12, 2.05 (s, 3H, CH_3Ac_). ^13^C NMR (CDCl_3_) δ 170.7, 170.0 (C_OAc_); 137.2 (C_Ar_); 128.5, 128.1, 128.0, 127.8 (C_Ar_); 106.2 (C_1_); 78.8 (C_4_); 77.5 (C_3_); 73.6 (C_2_); 73.2 (C_CH2_); 64.6 (C_5_); 55.0 (C_Me_); 20.8, 20.7 (C_MeAc_).

[α]_D_^20^= -9.3^o^ (c=0.1, CHCl_3_) HRMS *m/z* 356.171051 (calcd for C_17_H_26_O_7_N([M+NH_4_]^+^) 356.170928).

**Methyl 3-*O*-acetyl-5-*O*-*tert*-butyldimethylsilyl-β-D-ribofuranoside 5e**

^1^H NMR (CDCl_3_) δ 5.15 (dd, J_2,3_= 3.7 Hz, J_3-4_= 5.2 Hz, 1H, H_3_), 4.85 (s, 1H, H_1_); 4.24 (d, J_2,3_= 3.7 Hz, 1H, H_2_); 4.13 (ddd, J_4-5’_=5.6 Hz, J_4-5_=5.6 Hz, J_3-4_= 5.2 Hz, 1H, H_4_); 3.73, ABX (J_4-5_=5.6 Hz, J_5-5’_=11.1 Hz, 1H, H_5_); 3.68, ABX (J_4-5’_=4.6 Hz, J_5-5’_=11.1 Hz,1H, H_5’_) 3.37 (s, 3H, H_OMe_); 2.11 (s, 3H, CH_3Ac_); 0.88 (s, 9H, tBu); 0.07, 0.05 (s, 6H, CH_3_). ^13^C NMR (CDCl_3_) δ 170.2 (C_OAc_); 108.4 (C_1_); 81.4 (C_4_); 74.5 (C_3_); 74.3 (C_2_); 64.3 (C_5_); 55.3 (C_Me_); 25.8 (C_tBu_); 20.8 (C_MeAc_); 18.3 (C_Si_); -5.4, -5.5 (C_MeSi_).

**Methyl 2-*O*-acetyl-5-*O*-*tert*-butyldimethylsilyl-β-D-ribofuranoside 5f**

^1^H NMR (CDCl_3_) δ 5.01 (d, J_2,3_= 4.8 Hz, 1H, H_2_); 4.85 (s, 1H, H_1_); 4.41-4.38 (m, 1H, H_3_); 3.95 (ddd, J_4-5’_=4.9 Hz, J_4-5_=4.9 Hz, J_3-4_=6.6 Hz, 1H, H_4_); 3.75 (d, J_4,5’_= 4.9 Hz, 2H, H_5_, H_5’_); 3.34 (s, 3H, H_OMe_); 2.14 (s, 3H, CH_3Ac_); 0.90 (s, 9H, tBu); 0.07, 0.05 (s, 6H, CH_3_). ^13^C NMR (CDCl_3_) δ 170.5 (C_OAc_); 105.8 (C_1_); 83.3 (C_4_); 76.9 (C_2_); 71.3 (C_2_); 64.3 (C_5_); 55.1 (C_Me_); 25.9 (C_tBu_); 20.8 (C_MeAc_); 18.2 (C_Si_); -5.4, -5.5 (C_MeSi_).

**3-*O*-benzyl-1,2-*O*-isopropylidene-α-D-ribofuranoside 6**

Overall yield from xylose: 31.4% after 5 steps

^1^H NMR (CDCl_3_) δ 7.36-7.29 (m, 5H, H_Ar_); 5.70 (d, *J*_1,2_= 3.6 Hz, 1H, H_1_); 4.74 (AB, *J_A_*= 11.9 Hz, 1H, H_CH2_); 4.57 (AB, *J_A_*=11.9 Hz, 1H, H_CH2_); 4.54 (dd, *J_2,3_*= 4.3 Hz, *J_1,2_*= 3.6 Hz, 1H, H_2_); 4.11-4.07(m, 1H, H_4_); 3.88 (ABX, *J_4,5_*= 3.0 Hz, *J_5,5’_*= 12.5 Hz, 1H, H_5_); 3.82 (dd, *J_2,3_*= 4.3 Hz, *J_3,4_*= 9.1 Hz, 1H, H_3_); 3.61 (ABX, *J_4,5’_*= 1.8 Hz, *J_5,5’_*= 12.5 Hz, 1H, H_5’_); 2.22 (br, 1H, OH_5_); 1.57 (s, 3H, CH_3_); 1.34 (s, 3H, CH_3_). ^13^C NMR (CDCl_3_) δ 137.4 (C_Ar_); 128.3, 127.9, 127.8 (C_Ar_); 112.9 (C_qIso_); 103.9 (C_1_); 78.7 (C_4_); 77.4 (C_2_); 76.5 (C_3_); 72.2 (C_CH2_); 60.4 (C_5_); 26.7 (C_MeIso_); 26.4 (C_MeIso_). [α]_D_^20^=91.9^o^ (c=0.6, CHCl_3_) HRMS *m/z* 298.165109 (calcd for C_15_H_24_O_5_N([M+NH_4_]^+^) 298.165448).

**5-*O*-acetyl-3-*O*-benzyl-1,2-*O*-isopropylidene-α-D-ribofuranoside 7**

^1^H NMR (CDCl_3_) δ 7.36-7.31 (m, 5H, H_Ar_); 5.74 (d, *J_1,2_*= 3.7 Hz, 1H, H_1_); 4.78 (AB, *J_A_*=12.0 Hz, 1H, H_CH2_); 4.59 (dd, *J_2,3_*= 4.3 Hz, *J_1,2_*= 3.7 Hz, 1H, H_2_); 4.54 (AB, *J_A_*=12.0 Hz, 1H, H_CH2_); 4.33 (ABX, *J_4,5_*=5.1 Hz, *J_5,5’_*=12.2 Hz, 1H, H_5_); 4.31(ABX, *J_4,5’_*=2.3 Hz, *J_5,5’_*=12.2 Hz, 1H, H_5_); 4.23(ddd, *J_4,5’_*= 2.3 Hz, *J_4,5_*= 5.1 Hz, *J_3,4_*= 9.1 Hz, 1H, H_4_); 3.65 (dd, *J_2,3_*= 4.3 Hz, *J_3,4_*= 9.1 Hz, 1H, H_3_); 1.99 (s, 3H, CH_3Ac_); 1.60 (s, 3H, CH_3_); 1.37 (s, 3H, CH_3_). ^13^C NMR (CDCl_3_) δ 170.6 (C_OAc_); 137.2 (C_Ar_); 128.5, 128.1, 128.0 (C_Ar_); 113.1 (C_qIso_); 104.2 (C_1_); 77.3 (C_3_); 76.9 (C_2_); 76.2 (C_4_); 72.1 (C_CH2_); 62.8 (C_5_); 26.7 (C_MeIso_); 26.5 (C_MeIso_); 20.7 (C_MeAc_). [α]_D_^20^=105.7^o^ (c=0.9, CHCl_3_) HRMS *m/z* 340.174713 (calcd for C_17_H_26_O_6_N ([M+NH_4_]^+^) 340.176013).

**Acknowledgement:** The European Social Funds provided the financial support of O. Chevallier’s studentship.
